# Investigating the Behavior and Personality Structure of the Aldabra Tortoise during Human Interactions and Training Events

**DOI:** 10.3390/ani12040419

**Published:** 2022-02-10

**Authors:** Giovanni Quintavalle Pastorino, Vanessa Smith, Massimo Faustini, Eleonora Bonacina, Davide Guadagnini, Roberto Robbiati, Alice Cavalleri, James Edward Brereton, Richard Preziosi

**Affiliations:** 1 Zoo Biology, Manchester Metropolitan University, All Saints Building, All Saints, Manchester M15 6BH, UK; v_smith@hotmail.com (V.S.); cavallerialice@gmail.com (A.C.); 2 Università degli studi di Milano, Via Festa del Perdono, 7, 20122 Milan, Italy; massimofaustini@gmail.com (M.F.); ebonacina12@gmail.xom (E.B.); 3 Parco faunistico Le Cornelle, Via Cornelle, 16, 24030 Valbrembo, Italy; davideg@gmail.com (D.G.); r_robbiati@cornelle.com (R.R.); 4 Animal and Zoo Science, Higher Education, University Centre Sparsholt, Westley Lane, Sparsholt, Winchester SO21 2NF, UK; James.Brereton@sparsholt.ac.uk; 5 Higher Education, University of Plymouth, Drake Circus, Plymouth PL4 8AA, UK; richard.preziosi@plymouth.ac.uk

**Keywords:** *Aldabrachelys gigantea*, *Geochelone gigantea*, Chelonia, human–animal interaction, animal personality

## Abstract

**Simple Summary:**

There are many studies that suggest that a range of animal species have personalities, and that animals can benefit from interactions with their human caregivers. However, many of these previous studies have focused only on mammals, with fewer studies focusing on reptiles. Research was undertaken at the Faunistic Park Le Cornelle, Italy, to investigate the effects of approach tests and food interaction events on 5 male and 5 female Aldabra tortoises’ (*Aldrabrachelys gigantea*) behavior. There were differences in behavior between tortoises, and in response to different types of events. The tortoises also responded differently to their own keepers, vets, or unfamiliar people. The personality of the tortoises were also quantified using principal component analysis. Overall, the study revealed that individuals acted significantly differently to one another, and that while females initially appeared to display a greater number of shy behaviors, this was not consistent across the group. PCA revealed two personality dimensions in the tortoises, boldness and avoidance. Overall, the study revealed that the tortoises possessed personalities that were independent of sex or scenario. The study also revealed that many tortoises chose to interact with their keepers during training and approach tests: this suggests that human-tortoise interactions may have some enrichment value. Future research could quantify tortoise personality in other scenarios, such as in social interactions with other tortoises.

**Abstract:**

Human–animal interaction (HAI) can be valuable for captive animals, and many zoo-housed species benefit from interactions with their keepers. There is also an increasing body of evidence that some animal species possess personalities that are temporally consistent. However, the majority of zoo research, particularly on personality and HAI, traditionally has focused on mammals, and there are comparatively fewer studies on reptiles. Research was undertaken at the Faunistic Park Le Cornelle, Italy, to investigate the effects of approach tests and food interaction events on 5 male and 5 female Aldabra tortoise (*Aldrabrachelys gigantea*) behavior. During human–tortoise interactions, continuous focal sampling of behavior took place. The behavioral differences between individual tortoises, interaction type (approach or training) and person involved in the interaction (keeper, vet, or observer) were analysed using general linear mixed-effects models. The personality components of the tortoises were also quantified using principal component analysis. Overall, the study revealed that individuals acted significantly differently to one another, and that while females initially appeared to display a greater number of shy behaviors, this was not consistent across the group. PCA revealed two personality dimensions in the tortoises: boldness and avoidance. Overall, the study revealed that the tortoises possessed personalities that were independent of sex or scenario. The study also revealed that many tortoises chose to interact with their keepers during training and approach tests: this suggests that human–tortoise interactions may have some enrichment value. Future research could quantify tortoise personality in other scenarios such as in social interactions with other tortoises.

## 1. Introduction

Captive animals regularly come into contact with keeping staff or visitors. For humans, interaction with other species such as companion animals, may have benefits in terms of health, stress relief and immune function [1,2,3,4,5,6]. For animals, particularly non-domesticated species such as those housed in zoos, the relationship is often complex [2,4]. Some species may perceive any human presence to be a threat, similar to that of predators in their natural state. Others may consider the presence of visitors and keepers to be enriching [2,3,5]. Animals that consider human presence to be enriching, or at least non-threatening, are likely to cope better in a captive environment [4].

The study of animal personality has gained momentum in the zoo community in recent years, given its value in terms of animal compatibility and evolutionary function [3,4,5]. Personality in this case refers to responses to stimuli that are temporally stable and similar across different scenarios [4]. Personality in humans is well studied (for example, the five factor model), but the personality dimensions identified in humans cannot be directly applied to animals [5,6]. Research on animal personality has focused on mammalian species, particularly in the families of Primates [4,7,8], Suidae [9], Canidae [10,11] and Felidae [12,13], though also in fish [14]. Personality research has value in helping animal keepers to better understand the compatibility of animals in breeding programs, in applied research on the evolutionary development of personality [15,16], and in predicting conservation translocation success [17,18,19,20].

Traditionally, reptiles have received less research focus than mammals in terms of personality research [20], and in research in general [21]. However, reptiles are well represented in zoological collections [22], so there remains a research gap in the field of reptilian behavioral science. Historically, ectothermy was a barrier to behavioral research, as reptiles are generally inactive until they reach their preferred optimal temperature [22,23,24,25,26,27]. However, once sufficiently heated, reptiles have been shown to be capable of problem solving and social learning, equivalent to that of mammals [15].

While limited, there is some research available on tortoise personality [28]. Several studies have focused on the potential implications of personality on reintroduction outcomes and survival, particularly in the desert tortoise (*Gopherus agassizii*) [20,29]. In these studies, individuals that were more timid had a higher probability of survival in environments containing predators [29]. In the Hermann’s tortoise (*Testudo hermanni*), the personality dimensions of aggression and boldness were associated with darker shelled individuals [30,31]. At current, personality has been assessed for only a handful of tortoise species, and it is not clear if the personality dimensions that have been identified are consistent across the taxon.

The Aldabra giant tortoise (*Aldabrachelys* (*Geochelone*) *gigantea*) is one of the world’s largest tortoise species, with some individuals reaching weights of more than 250 kg [32]. Endemic to the Aldabra Atoll, the species is described as vulnerable by the International Union for the Conservation of Nature [33], and has been used in conservation translocations to the Seychelles to replace extinct tortoise species [34]. In the wild, *A. gigantea* act as a keystone species, grazing and transporting the seeds of a wide range of native shrubs, grasses, and leaves [35]. There is a well-marked daily cycle of activity, with feeding being limited to the early morning and late evening [34]. Wild social groupings and population densities are variable, and tortoises may be found on their own or may congregate into small herds depending on food availability [32,36]. Agonistic behavior is reported to be virtually absent among wild groups [32]. The species is of conservation concern due to historic collection of tortoises as a food source and competition with goats for grazing [36,37]. The zoological community has recognized the threats to the wild population and have bred this species in captivity. For example, a review of Species360’s Zoological Information Management System (ZIMS) revealed over 800 Aldabra giant tortoises in captivity at the time of writing [38].

Given the large number of individuals in captivity, along with the potential conservation value of captive specimens, the Aldabra giant tortoise is a good candidate for behavioral research [25,39]. Research may also allow *A. gigantea* to act as a model species, promoting further personality studies on a range of Chelonia species. Given the limited published research available on human–animal interactions for reptiles [25,40], this species has potential as a useful study subject.

The aim of this study was to investigate the personality structure of zoo-housed Aldabra giant tortoises during food interaction events. Observations of behavioral responses during these events were used to identify whether individual tortoises were consistent in their responses to animal care staff during training events.

## 2. Methods

### 2.1. Study Subjects

**Figure 1:**
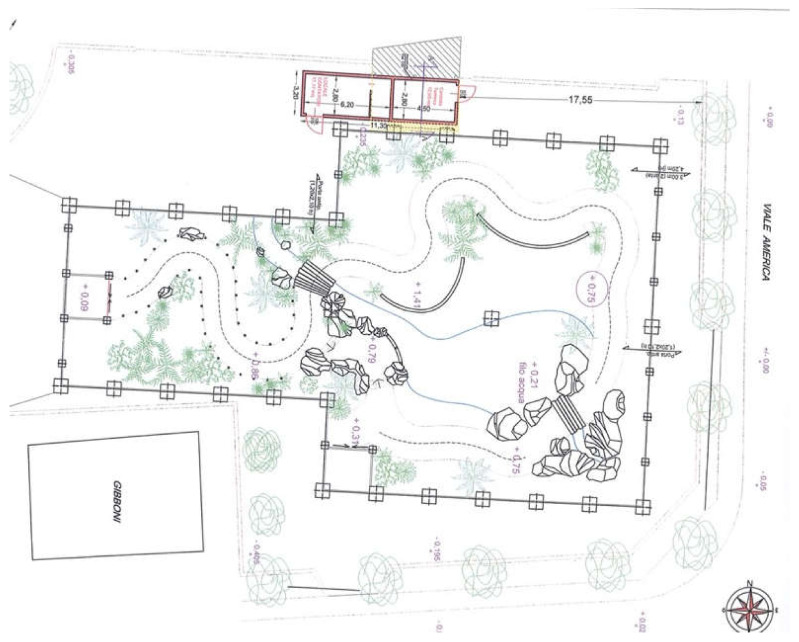
Enclosure design for the tortoise group.

Before the study was undertaken, the project was ethically reviewed and approved by the Ethical Review Committee at Manchester Metropolitan University. The study was carried out on 5 male and 5 female Aldabra tortoises, which were housed at the Faunistic Park Le Cornelle in Valbremo, Italy. A picture identification guide was produced before the study, allowing individuals to be identified based on morphological features. Individuals were also referred to by names they were given, throughout the study (Table 1). All tortoises were mature adults, each with an estimated age between 30–60 years. The tortoises were housed in an indoor exhibit 840.38 m^2^, which was open to the public from 10:00 until 17:00 (Figure 1). Tall barriers prevented the tortoises from interacting with the public. Research was undertaken in the indoor exhibit where the temperature was kept between 20–27 °C to mimic the tortoises’ natural climate [25].

### 2.2. Data Collection

Between 10 and 16 approach tests were carried out on each tortoise (with variance due to tortoise interest in the trials), with four different people undertaking the tests. These consisted of two keepers (known to the tortoises), a vet and an unknown observer. The slow movements of tortoises, combined with time restrictions, limited the number of replicates that could take place. Each test began with a person standing 5 m away from one of the tortoises and slowly walking towards them, stopping beside the individual. After a period of 30 sec, the person would crouch down and rub the tortoise shell, head, and legs. Similar interactions have been conducted by other researchers on the Aldabra tortoise [25], and other tortoise species [26,40].

Food interaction events were also performed with each tortoise. Between two and five training events were conducted per animal, with variance in the number of trials occurring because of differences in engagement levels between tortoises. Food interaction events consisted of one of the keepers approaching and presenting the individual with a piece of food speared onto the end of a stick. Gradually, as the tortoise became more comfortable, the keeper would move further away from the tortoise, encouraging them to step forward and take the piece of food. The food items presented to them included grapes (*Vitis vinifera*), apple (*Malus domestica*), sweet pepper (*Capsicum annuum*) and plum (*Prunus domestica*): these items were selected because they were considered attractive to the tortoises. Previous research has shown that tortoises show a preference for red, yellow, and orange food items [40,41]. The food items used in food interaction events were not given as part of the normal diet of the tortoises which encouraged them to cooperate. Tortoises were free to walk away from the social interaction at any point during the approach tests and food interaction events.

All approach tests and food interaction events were video recorded by the same researcher, who kept still and was partly concealed by the exhibit perimeter. This individual conducted all observations and conducted analysis of all videos.

Prior to the study, an ethogram of the Aldabra tortoises at Faunistic Park Le Cornelle was constructed for use in a pilot study, adapted from Ruby and Niblick’s [42] ethogram. After initially reviewing the video data, the ethogram was adapted, taking the most relevant social and defensive behaviors from the original ethogram (Table 2). Behaviors were categorized as either “fearful” or “bold” in response to human interaction.

Each video was analysed using Behavioral Observation Research Interactive Software (BORIS) [43]. Continuous focal sampling was used to record all the behaviors exhibited by each individual during approach tests and food interaction events. Total video length for each individual varied greatly, for both approach tests and food interaction events. Therefore, the analysed data were exported to Microsoft Excel where the durations of state behaviors were converted into percentages of time and events were converted into counts per minute.

### 2.3. Data Analysis

All data were uploaded into a Microsoft Excel 2013™ spreadsheet. Statistical analyses were performed using Minitab version 17. To analyze the behavioral impact of the approach tests and food interaction events, general linear mixed-effects models (GLMM) were run. Both the approach tests and food interaction events were combined into one single set of GLMMs. In these models, the behavior was included as the outcome, and the session type (food interaction events or approach), the individual tortoise and the person present (keeper, vet, or unknown observer) were used as predictors. The sex of the tortoise was initially used as a predictor, but due to its high collinearity with the individual, it was discarded.

Principal component analysis (PCA) was then used to illustrate how individuals differed from one another in their response to human interaction, both during approach tests and food interaction events [44,45]. PCA is used to determine which variables are commonly correlated, and to reduce a range of variables into a smaller number, known as components. For the PCA, all behaviors were input into the analysis (excluding food bite and food sniff, which were both zero inflated). Individual differences in behavioral response to approach tests and food interaction events were tested for normality. As all were identified as being not normally distributed, the behaviors were examined using Kruskal–Wallis tests and further visualized using PCA.

## 3. Results

### 3.1. Behavior

**Figure 2:**
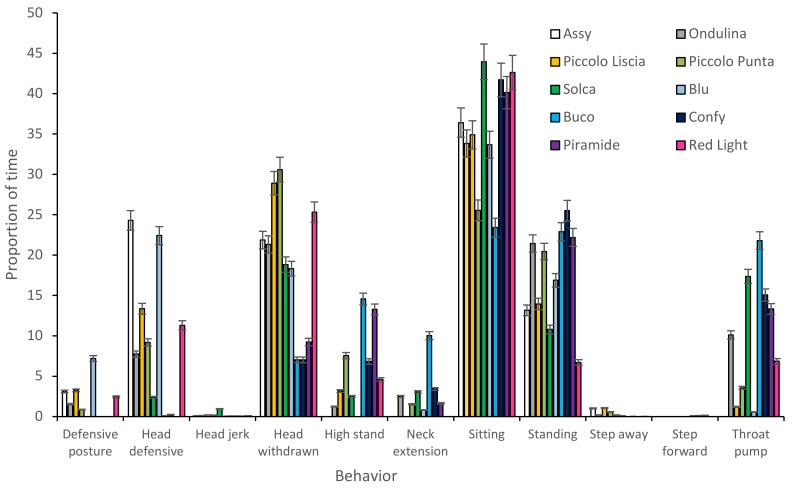
Difference in Aldabra tortoise behavior during approach tests (+/−standard error).

**Figure 3:**
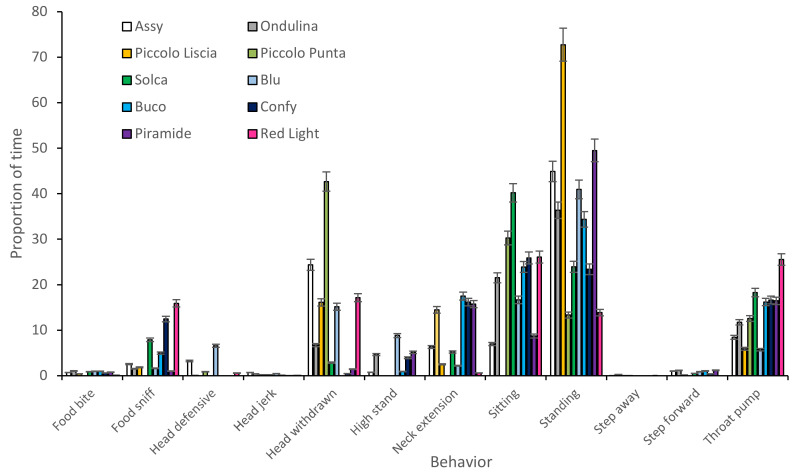
Difference in individual Aldabra tortoises’ behavior during food interaction events (+/−standard error).

An initial graph was produced to show the difference between the behavior of individual tortoises during the approach tests (Figure 2). Two behaviors, food bite and food sniff, were removed as they were not relevant to the approach tests. Graphs were produced to illustrate differences between individual tortoises (Figure 3). As in the approach tests, individual tortoises showed considerable differences between each other in terms of their behavior during food interaction events.

GLMM modelling for the approach tests and food interaction events combined, found that at least one predictor was significant for each behavior outcome, and often all three predictors were significant (Table 3). All models were significant. However, some models explained the variance in behavior better than others: *R*^2^ values varied from 8.94% (head jerk) to 41.42% (head withdrawn). The individual tortoise was a significant predictor of all behaviors except sitting, standing, and stepping forward.

### 3.2. Personality

**Figure 4:**
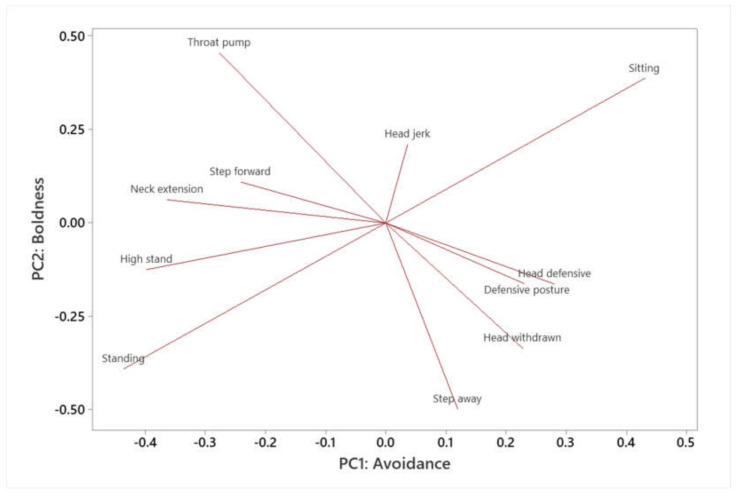
Output of PCA of Aldabra tortoise behavior during approach tests. A total of two components, labelled ‘boldness’ and ‘avoidance’, were identified.

**Figure 5:**
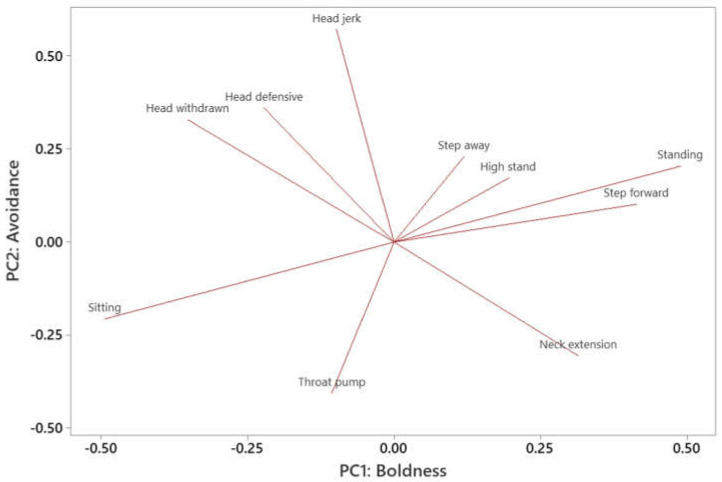
Output of PCA of Aldabra tortoise behavior during food interaction events. The two principal components, labelled ‘boldness’ and ‘avoidance’, were similar to those identified in approach tests.

PCA of individual tortoise responses to approach tests and food interaction events were conducted (Figure 4 and Figure 5). Behaviors were inputted into the PCA to identify which behaviors occurred most commonly together. 

In the PCA for approach tests, a total of 11 components were identified. As PC1 and PC2 explained the majority of the variance, they were selected and the remaining components were discarded. PC1 and PC2 explained 30.2% and 15.9% of the variance respectively (Figure 4). In total, 46.1% of the total variance was explained using the PCA (Table 4 and Table 5). For PC1, the behaviors that showed the most positive loading were ’Step away’, ‘High stand’, ‘Head defensive’, ‘Defensive posture’ and ‘Neck extension’. These behaviors appear to be involved in avoidance or defense. ‘Throat pump’, ‘High stand’ and ‘Step forward’ were negatively loaded on PC1. Higher values of PC1 therefore indicate individuals that are more averse to interaction: to take this into account, PC1 was labelled as ‘Avoidance’. 

Higher values of PC2 were indicative of more confident individuals. ‘Throat pump’, ‘High stand’ and ‘Step forward’ were all negatively loaded on PC2. The behaviors ‘Defensive posture’, ‘Head defensive’ and ‘Step away’, by contrast, were negatively loaded on PC2. Pirimide, Bucco and Confy appeared to be the most bold, with Assy, Blu and Piccolo Liscia being the most fearful during approach tests. PC2 was labelled as ‘boldness’.

For the food interaction events (Figure 5), 10 Principal Components were identified. PC1 and PC2 explained 28.4% and 20.2% of the variance respectively: 48.7% of variance was explained in total (Table 6 and Table 7). As a result, the first two components were utilized. The behaviors were loaded in a similar fashion to those identified in the approach tests, though in this case, PC1 was positively loaded with bold behaviors (e.g., neck extension, step forward, standing) and PC2 was associated with avoidance behaviors (head jerk, head defensive, head withdrawn). As a result, this time PC1 was labelled as ‘boldness’ and PC2 was labelled as ‘avoidance’. 

## 4. Discussion

Overall, the study revealed that Aldabra tortoises reacted differently to the approach tests and training events. While the behavioral differences initially appeared to be related to sex, the differences were better explained by differences between individual tortoises. Two personality dimensions: boldness and avoidance, were identified using PCA.

### 4.1. Tortoise Behavior and Personality

On first inspection, there appeared to be considerable sex related differences in behavior during training and approach tests. Namely, male tortoises appeared to spend more time engaged in high stands, food sniffing, neck extensions and throat pumping. All of the above behaviors tend to suggest a more confident individual. For example, extension of the neck leaves the animal in a more vulnerable position; a state avoided in shyer individuals [42]. In contrast, females were more likely to spend time sitting, withdrawing their heads, or adopting a defensive posture. These behaviors generally suggest a more defensive or shy temperament.

However, closer inspection of the data and statistical analysis showed this not to be the case: whilst on average female tortoises engaged in more defensive behaviors, this was not consistent across all individuals in the group. For example, there were some females that rarely took up the ‘head defensive’ posture, and some males that spend long periods of time engaged in this behavior. This suggests that analysis at the individual level is a better method of assessing temperament [26,27].

The first personality dimension identified in the study was boldness. Boldness was associated with behaviors such as extension of the neck, high stand, and step forward. Boldness has also been identified as a personality dimension in other chelonians, such as the Hermann’s [31] and desert tortoise [17], and eastern box turtles (*Terrapene carolina*) [46]. Boldness has a survival context for wild tortoises: in some studies, desert tortoises were more likely to be predated if they expressed a bold personality, as they were more likely to explore and come across coyotes (*Canis latrans*) [20,28]. In contrast, boldness may have a survival advantage in environments where predators are scarce, as bolder animals may have more opportunities to find food and mates than their shy counterparts [20], or maintain a higher body temperature [45]. In the wild, there are few natural predators of adult Aldabra tortoises [32], so in theory a bolder temperament may come with advantages. However, bold individuals may also be more likely to explore new, unsuitable terrain. A mix of both bold and shy individuals in the population may therefore be beneficial.

The PCA also identified one other personality dimension, which was labelled here as avoidance. Throat pumping was positively loaded on this dimension, whereas avoidance behaviors such as ‘step away’ and ‘high stand’ were negatively loaded. Behaviors such as biting, and ramming were not observed in the study because individuals were not observed interacting with one another. However, behaviors such as throat pumping associated with confidence and lack of avoidance in tortoise species [42]. Boldness and avoidance are not mutually exclusive: a tortoise could be both shy and averse to interactions, or non-avoiding and bold. In this study, an individual that showed a low aggression score tended to avoid interaction with keepers, veterinarians and observers.

Aggression has an environment-dependent survival advantage. In environments where other tortoises are non-aggressive, an aggressive individual may be more successful in competing for food resources or mates. In contrast, an aggressive individual in an aggressive tortoise community is more likely to engage in fights that could result in injury [28]. Previous studies in Hermann’s tortoises have showed that aggression was correlated with darker shell colors [30]. Whilst no such research has been conducted on Aldabra tortoise’s, it appears that aggression/avoidance is a personality dimension for both species.

It is beyond the scope of this study to identify the origin of each tortoise personality. Previous research in reptiles suggests that the personality of some species may be affected by egg incubation temperature [47]. Rearing and the environment of the tortoise may also influence the personality [20]. As they are a long-lived species, the individual life history of each tortoise observed may have influenced their behavior during interactions with people.

There is some practical value in assessing the personality of captive Aldabra tortoises. Understanding individual temperaments may allow keepers to better predict how individuals will interact when provided with novel enrichment types. Similarly, personality assessment might be used when planning to move animals for the purpose of breeding: bold, aggressive individuals could pose a challenge when group-housed alongside shy, non-aggressive individuals. Social dynamics vary between tortoise populations and species; social dynamics therefore need to be assessed within individual populations.

### 4.2. Human–Tortoise Interaction

The discovery that some tortoises may choose to interact with humans, and find the process enriching, is still a relatively novel finding [26,27,40]. During this study, individual tortoises had the ability to walk away from human interactions, yet some individuals spent long periods of time with keepers and appeared to enjoy the interaction. Tortoises were also able to differentiate between people: the person present was a significant predictor of defensive posture, food sniff and high stand. It is possible, therefore, that tortoises were able to recognize either the person (or the clothing) of their keepers versus the vet or unknown observer. It should be noted, however, that as only one vet and keeper were available, it is not known whether the tortoises recognized the person or the clothing. Generally, defensive behaviors were observed more when the vet was present: this may be a learned response as a result of previous unpleasant interactions during veterinary care or treatment. Behaviors such as the high stand and food sniff occurred more when keepers were present. This suggests that tortoises may both be able to identify individuals (or their clothing) and remember the results of prior interactions.

### 4.3. Future Directions

Whilst the sample size for this study was good, all tortoises were housed in the same collection. Potential husbandry effects on tortoise behavior could be accounted for in a multi-zoo study [48]. Similarly, temperature is known to affect tortoise behavior [24,28]. Whilst the temperature within the enclosure was considered suitable for Aldabra tortoises, individual temperature assessments could be used to determine whether tortoises were more likely to interact with training when their body temperatures were higher [27].

Personality assessments in animals have been conducted for a range of species, including primates, carnivores, birds, and to a lesser extent, reptiles [30,49]. Two general methodologies exist for assessing animal temperament: the first involves observing the animal under a range of scenarios to determine whether their behavior is temporally consistent. The second technique involves asking caretakers to assess each animal’s temperament [3]. When used together, keeper responses can be correlated against observed behavior to investigate how effective keepers are in assessing animal personality. This study used only behavioral observations: future research could include keeper questionnaires in order to validate personality elements.

Future studies could also extend this research question to determine whether tortoises are able to discriminate between individuals, or whether they are only able to identify individuals based on more generic features (such as clothing). It was beyond the scope of this study to identify whether the tortoises could identify individuals if they were not dressed in their normal clothing (e.g., keeper, vet). This could be incorporated into a future study.

One final direction is to investigate the social interactions between individual Aldabra tortoises. It was beyond the scope of this study to investigate intra-specific interactions as tortoises were trained individually. Given the plasticity in social grouping of Aldabra tortoises in the wild, it is possible that many affiliative interactions exist, which would not be picked up by this study’s methodology [36,37]. Future studies could incorporate a social network component to better understand Aldabra tortoise sociality.

## 5. Conclusions

Over the course of this study two key messages were identified. Firstly, two personality dimensions were identified during the study: boldness and avoidance of interactions. Whilst this study only considered one group of Aldabra tortoises in one zoo, the findings are similar to those for Hermann’s tortoises and gopher tortoises in both wild and captive settings. Extrapolating from these results, it is possible that these personality dimensions are conserved across the Chelonia taxonomic group: further research on more tortoise species would be beneficial.

Secondly, whilst there was variance in behavior between individuals, some Aldabra tortoises chose to interact with people, even when there was no reward involved. Given these results, it is possible that keeper interaction is a viable enrichment tool for those working with Aldabra tortoises.

## Figures and Tables

**Table 1:** Aldabra tortoise individuals.

Tortoise ID	Sex	Description
Assy	Female	Medium, smooth, grey carapace
Blu	Male	Medium, smooth grey carapace
Bucco	Male	Large, rough, red carapace
Confy	Male	Large, rough, red carapace
Ondulina	Female	Medium, smooth, grey carapace
Piccolo Liscia	Female	Small, smooth, grey carapace (smallest individual)
Piccolo Punta	Female	Small, smooth, grey carapace
Pirimide	Male	Large, rough, red carapace (largest individual)
Red Light	Male	Large, rough, red carapace
Solca	Female	Medium, smooth, grey carapace

**Table 2:** Aldabra tortoise ethogram. Adapted from Ruby and Niblick [42]. States are shown using ^s^ and events as ^e^.

Behavior	Description
Defensive posture ^s^	Head and forelegs tucked in tightly, with back legs extended pushing front of body downwards. Only recorded during approach tests.
Head defensive ^e^	Head fully withdrawn into shell with limbs still exposed.
Head jerk ^e^	Sudden withdrawal of the head, either by shortening the neck or retreating head into shell.
Head withdrawn ^s^	Head withdrawn but still visible. Neck retracted and not exposed.
Sitting ^s^	Body resting on ground with all limbs exposed.
Step away ^e^	Tortoise steps away from person present.
Neck extension ^s^	Neck fully extended, reaching forward.
Standing ^s^	Body raised on all four limbs but close to the ground.
High stand ^s^	Legs fully extended with body raised fully off of the ground.
Step forward ^e^	Tortoise steps towards the person present.
Throat pump ^s^	Clear, steady pulsation of the neck, more obvious when neck is extended.
Food sniff ^e^	Nose exploration of the food item, close to or direct contact. Only recorded during training sessions
Food bite ^e^	Tortoise bites the piece of fruit offered on a stick. Only recorded during training sessions

**Table 3:** Outputs of General Linear Models (GLMM) on tortoise behavior during food interaction events. For the predictor of ‘person present’, the SE coefficients are displayed as pairwise comparisons against animal keeper 1. K1 = Keeper 1, K2 = Keeper 2, V = Vet, U = Unknown person. * indicates a staistically significant variable.

Behavior	*R^2^* (*p*)	Predictor	DF	SE Coefficient	*p*
Defensive posture	27.38% (*p* < 0.001 *)	Session type	1	1.58	0.009 *
		Person present	3	K1-K2: 1.63, K1-V: 1.65, K1-U: 1.58	0.038 *
		Individual tortoise	9	2.30	<0.001 *
Food bite	36.68% (*p* < 0.001 *)	Session type	NA	NA	NA
		Person present	3	K1-K2: 0.10, K1-V: 0.10, K1-U: 0.09	<0.001 *
		Individual tortoise	9	0.166	0.031 *
Food sniff	18.66% (*p* < 0.001 *)	Session type	NA	NA	NA
		Person present	3	K1-K2: 1.31, K1-V: 1.26, K1-U: 1.34	<0.001 *
		Individual tortoise	9	2.18	0.007 *
Head defensive	39.28% (*p* < 0.001 *)	Session type	1	4.40	<0.001 *
		Person present	3	K1-K2: 3.99, K1-V: 3.85, K1-U: 4.07	0.027 *
		Individual tortoise	9	6.63	<0.001 *
Head jerk	8.94% (*p* = 0.010 *)	Session type	1	0.28	0.332
		Person present	3	K1-K2: 0.24, K1-V: 0.24, K1-U: 0.23	0.138
		Individual tortoise	9	0.335	0.008 *
Head withdrawn	41.42% (*p* < 0.001 *)	Session type	1	5.91	0.269
		Person present	3	K1-K2: 6.07, K1-V: 5.91, K1-U: 6.17	0.017 *
		Individual tortoise	9	8.58	<0.001 *
High stand	16.90% (*p* < 0.001 *)	Session type	1	4.64	0.329
		Person present	3	K1-K2: 4.77, K1-V: 4.65, K1-U: 4.85	0.005 *
		Individual tortoise	9	6.74	0.001 *
Neck extension	36.55% (*p* < 0.001 *)	Session type	1	2.67	0.001 *
		Person present	3	K1-K2: 2.75, K1-V: 2.68, K1-U: 2.86	0.201
		Individual tortoise	9	3.88	0.001 *
Sitting	16.33% (*p* < 0.001 *)	Session type	1	8.49	0.001 *
		Person present	3	K1-K2: 8.73, K1-V: 8.51, K1-U: 8.78	0.144
		Individual tortoise	9	12.30	0.276
Standing	12.32% (*p* < 0.001 *)	Session type	1	8.42	0.004 *
		Person present	3	K1-K2: 8.66, K1-V: 8.44, K1-U: 8.80	0.155
		Individual tortoise	9	12.20	0.151
Step away	27.00% (*p* < 0.001 *)	Session type	1	0.274	0.303
		Person present	3	K1-K2: 0.281, K1-V: 0.274, K1-U: 0.286	0.587
		Individual tortoise	9	0.397	0.001 *
Step forward	28.25% (*p* < 0.001 *)	Session type	1	0.178	0.001 *
		Person present	3	K1-K2: 0.183, K1-V: 0.186, K1-U: 0.178	0.628
		Individual tortoise	9	0.258	0.152
Throat pump	29.89% (*p* < 0.001 *)	Session type	1	4.81	0.036 *
		Person present	3	K1-K2: 4.95, K1-V: 4.82, K1-U: 5.03	0.086
		Individual tortoise	9	6.99	0.001 *

**Table 4:** Output of principal component analysis for approach tests. A total of 11 Principal Components were identified.

	PC1	PC2	PC3	PC4	PC5	PC6	PC7	PC8	PC9	PC10	PC11
Eigenvalue	3.3173	1.7516	1.2401	1.0241	0.9310	0.7366	0.5969	0.5225	0.4835	0.3654	0.0311
Proportion	0.302	0.159	0.113	0.093	0.085	0.067	0.054	0.048	0.044	0.033	0.003
Cumulative	0.302	0.461	0.574	0.667	0.751	0.818	0.872	0.920	0.964	0.997	1.000

**Table 5:** Eigenvector values for PCA and PC2 for approach tests.

Variable	PC1 (Avoidance)	PC2 (Boldness)
Sitting	0.431	0.387
Head defensive	0.28	−0.163
Defensive posture	0.23	−0.162
Head withdrawn	0.228	−0.336
Step away	0.12	−0.498
Head jerk	0.036	0.211
Step forward	−0.241	0.11
Throat pump	−0.277	0.456
Neck extension	−0.364	0.062
High stand	−0.398	−0.125
Standing	−0.436	−0.391

**Table 6:** Output of principal component analysis for food interaction events. A total of ten principal components were identified.

	PC1	PC2	PC3	PC4	PC5	PC6	PC7	PC8	PC9	PC10
Eigenvalue	2.8444	2.0226	1.1036	1.0776	0.8430	0.7685	0.5263	0.4949	0.2526	0.0664
Proportion	0.284	0.202	0.110	0.108	0.084	0.077	0.053	0.049	0.025	0.007
Cumulative	0.284	0.487	0.597	0.705	0.789	0.866	0.919	0.968	0.993	1.000

**Table 7:** Eigenvector values for PCA and PC2 for food interaction events.

Variable	PC1 (Boldness)	PC2 (Avoidance)
Standing	0.489	0.204
Step forward	0.414	0.102
Neck extension	0.315	−0.306
High stand	0.196	0.173
Step away	0.119	0.23
Head jerk	−0.098	0.571
Throat pump	−0.106	−0.406
Head defensive	−0.222	0.361
Head withdrawn	−0.352	0.329
Sitting	−0.493	−0.206

## Data Availability

Data can be requested through correspondence with the corresponding author. The data are not publicly available due to permissions from the zoological collection.
